# Impact of Cardiopulmonary Resuscitation of Donors on Days Alive and Out of Hospital after Orthotopic Heart Transplantation

**DOI:** 10.3390/jcm11133853

**Published:** 2022-07-03

**Authors:** Sebastian Roth, René M’Pembele, Anthony Nucaro, Alexandra Stroda, Theresa Tenge, Giovanna Lurati Buse, Stephan U. Sixt, Ralf Westenfeld, Philipp Rellecke, Igor Tudorache, Markus W. Hollmann, Hug Aubin, Payam Akhyari, Artur Lichtenberg, Ragnar Huhn, Udo Boeken

**Affiliations:** 1 Department of Anesthesiology, Medical Faculty and University Hospital Duesseldorf, Heinrich-Heine-University Duesseldorf, 40225 Duesseldorf, Germany; sebastian.roth@med.uni-duesseldorf.de (S.R.); rene.mpembele@med.uni-duesseldorf.de (R.M.); annuc100@hhu.de (A.N.); alexandra.stroda@med.uni-duesseldorf.de (A.S.); theresa.tenge@med.uni-duesseldorf.de (T.T.); giovanna.luratibuse@med.uni-duesseldorf.de (G.L.B.); stephanurs.sixt@med.uni-duesseldorf.de (S.U.S.); ragnar.huhn@med.uni-duesseldorf.de (R.H.); 2 Department of Cardiology, Pulmonology and Vascular Medicine, Medical Faculty and University Hospital Duesseldorf, Heinrich-Heine-University Duesseldorf, 40225 Duesseldorf, Germany; ralf.westenfeld@med.uni-duesseldorf.de; 3 Department of Cardiac Surgery, Medical Faculty and University Hospital Duesseldorf, Heinrich-Heine-University Duesseldorf, 40225 Duesseldorf, Germany; philipp.rellecke@med.uni-duesseldorf.de (P.R.); igor.tudorache@med.uni-duesseldorf.de (I.T.); hug.aubin@med.uni-duesseldorf.de (H.A.); payam.akhyari@med.uni-duesseldorf.de (P.A.); udo.boeken@med.uni-duesseldorf.de (U.B.); 4 Department of Anesthesiology, Amsterdam University Medical Center (AUMC), Location AMC, 1105 AZ Amsterdam, The Netherlands; m.w.hollmann@amsterdamumc.nl; 5 Department of Anesthesiology, Kerckhoff Heart and Lung Center, 61231 Bad Nauheim, Germany

**Keywords:** heart failure, heart transplantation, cardiopulmonary resuscitation, patient centered outcomes, quality of life, mortality

## Abstract

Background: The number of patients waiting for heart transplantation (HTX) is increasing. Optimizing the use of all available donor hearts is crucial. While mortality seems not to be affected by donor cardiopulmonary resuscitation (CPR), the impact of donor CPR on days alive and out of hospital (DAOH) is unclear. Methods: This retrospective study included adults who underwent HTX at the University Hospital Duesseldorf, Germany from 2010–2020. Main exposure was donor-CPR. Secondary exposure was the length of CPR. The primary endpoint was DAOH at one year. Results: A total of 187 patients were screened and 171 patients remained for statistical analysis. One-year mortality was 18.7%. The median DAOH at one year was 295 days (interquartile range 206–322 days). Forty-two patients (24.6%) received donor-CPR hearts. The median length of CPR was 15 (9–21) minutes. There was no significant difference in DAOH between patients with donor-CPR hearts versus patients with no-CPR hearts (CPR: 291 days (211–318 days) vs. no-CPR: 295 days (215–324 days); *p* = 0.619). Multivariate linear regression revealed that there was no association between length of CPR and DAOH (unstandardized coefficients B: −0.06, standard error: 0.81, 95% CI −1.65–1.53, *p* = 0.943). Conclusions: Donor CPR status and length of CPR are not associated with reduced DAOH at one year after HTX.

## 1. Introduction

The number of patients waiting for heart transplantation (HTX) is constantly increasing due to factors such as demographic shift and improved medical treatment [1,2,3,4,5,6,7,8]. The number of available donor hearts, however, does not match the high demand for these organs. According to Eurotransplant’s annual report for Germany, 329 donor hearts were transplanted in the year 2021, while 727 patients remained on the waiting list for HTX at the end of the year [9]. To maximize the benefit from available donor organs, it is crucial to optimize the allocation of potential donor hearts.

One criterion to consider in the allocation is a status of cardiopulmonary resuscitation (CPR) of the donor. Recent studies showed that mortality was not altered by the usage of donor CPR hearts after HTX, even when adjusted for longer durations of CPR and no-flow-time [10,11,12,13]. However, from a patient point of view, there might be other important factors next to solely survive and it is unclear how donor CPR affects patient quality of life. Days alive and out of hospital (DAOH) has been suggested as an alternative endpoint to quantify life impact, as it captures mortality, re-hospitalizations, and quality of life to an extent [14,15,16,17]. In this study, we evaluated the impact of donor CPR on DAOH in patients undergoing HTX. Our primary hypothesis was that, consistent with the existing mortality data, there might be no difference in DAOH after HTX when donor-CPR hearts were used compared with donor hearts without CPR. Another objective was to analyze the effect of CPR length on DAOH.

## 2. Materials and Methods

This study was conducted as a retrospective cohort study at the University Hospital Duesseldorf in accordance with the declaration of Helsinki and the guidelines for good clinical practice. The ethical review board of the Heinrich Heine University Duesseldorf approved the study protocol (reference number 4567). As all patients gave their written informed consent to be included in the prospective heart transplantation database of the University Hospital Duesseldorf, the need for additional written informed consent for this retrospective analysis could be waived. The present analysis complements a recent analysis by M’Pembele et al. (under review) which investigated life impact of perioperative variables after HTX. All included variables in this study were based on a meta-analysis [18]. As donor-CPR was not included into this meta-analysis (and consequently not included into the study), this separate analysis was performed.

This report was written according to the “Strengthening the Reporting of Observational studies in Epidemiology” (STROBE) guidelines [19].

### 2.1. In- and Exclusion Criteria/Study Participants

Inclusion criteria for this study were defined as HTX from September 2010 to December 2020 at our institution and age ≥18 years. Exclusion criteria were: incomplete medical records and missing data regarding the main exposures and/or the primary endpoint. Patients were then divided into two groups according to their main exposure: patients receiving hearts of donors that underwent cardiopulmonary resuscitation (donor-CPR-group) and patients receiving CPR-naive hearts (no-CPR-group). As a secondary exposure, we analyzed the length of CPR. All data were extracted from the local HTX database, as well as from electronic medical charts and included patient characteristics, medical history, and hospitalizations within one year.

### 2.2. Measurement of Endpoint DAOH

The primary endpoint of this study was days alive and out of hospital (DAOH) at one year after HTX. The calculation of DAOH was performed in the same manner as reported previously [16,17]. Briefly, DAOH is equal to the sum of days in hospital for one patient, subtracted from 365 days. In the case that a patient did not survive until one year after HTX, the difference between days survived and 365 days was added to the sum of days in hospital before subtraction from 365. Hospitalizations were defined as planned or unplanned stays of at least one day in hospital. As all HTX patients are very closely connected to our center, it is very unlikely that there were external hospitalizations without our knowledge. As a secondary endpoint, we analyzed mortality at one year after HTX to oppose this endpoint with DAOH.

### 2.3. Statistical Analysis

Statistical analysis was performed using GraphPad Prism© (Version 8.02, LaJolla, CA, USA) and IBM SPSS^©^ (Version 26.0, Armonk, NY, USA). Continuous variables are reported as mean ± standard deviation (SD) or as median with interquartile ranges (IQR) whenever appropriate, while categorical variables are presented as absolute numbers and percentages.

To compare DAOH depending on the donor-CPR status, we performed a Mann–Whitney U test. In order to assess the impact of the CPR length, we stratified the donor-CPR group by quartiles of CPR duration in four groups (<9 min, 9–14 min, 15–21 min, and >21 min) of similar size. Group comparison was performed with a Kruskal–Wallis test adjusted for multiple comparisons. Further, we conducted univariable linear regression to quantify the potential correlation between donor-CPR duration and DAOH. This was expanded by a multivariate linear regression model adjusting for donor age, mechanical ventilation, and renal replacement therapy based on M’Pembele et al. 2022 (under review). Finally, univariate survival analysis of donor-CPR- and no-CPR patients was conducted by computing Kaplan–Meier curves.

## 3. Results

**Figure 1:**
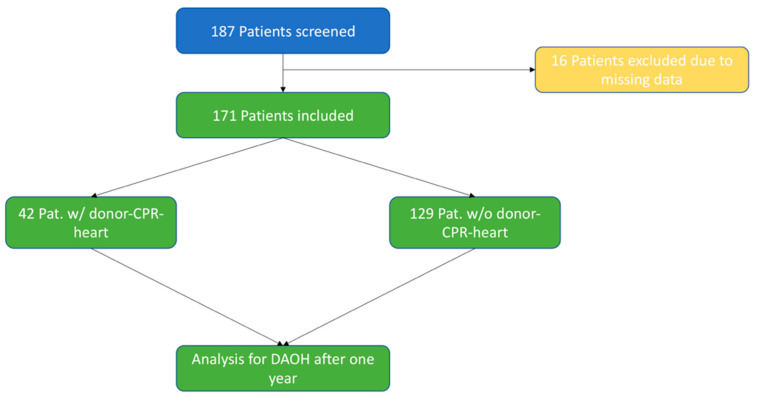
Study flow chart.

In total, 187 patients underwent HTX at out center from September 2010 to December 2020. After exclusion of 16 (6.4%) patients due to missing data regarding CPR length or DAOH, 171 eligible patients were identified and analyzed, of which 42 (24.6%) received hearts from donors that underwent cardiopulmonary resuscitation (see Figure 1).

Mean age for recipients was 54 ± 11 years, 74 out of 171 (43%) patients were female. Mean age for donors was 43 ± 13 years. The median length of CPR was 15 min (IQR: 9–21 min). The inotropic dobutamine was administered to 21% of the donors with CPR status and to 9% of the no-CPR donors. Recipients and donor characteristics are specified in detail in Table 1. Overall, 32 (18.7%) patients had died after one year. Median DAOH after one year for the entire cohort was 295 (interquartile range (IQR) 206–322 days).

**Figure 2:**
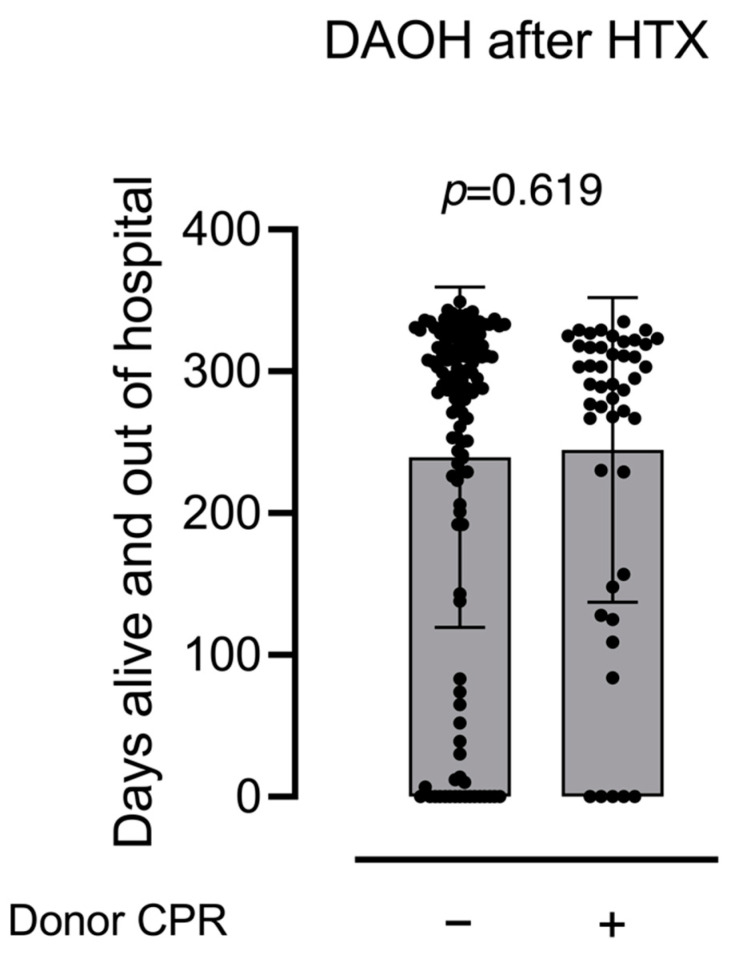
Comparison of days alive and out of hospital at one year after heart transplantation between patients who received donor hearts with and without history of cardiopulmonary resuscitation.

**Figure 3:**
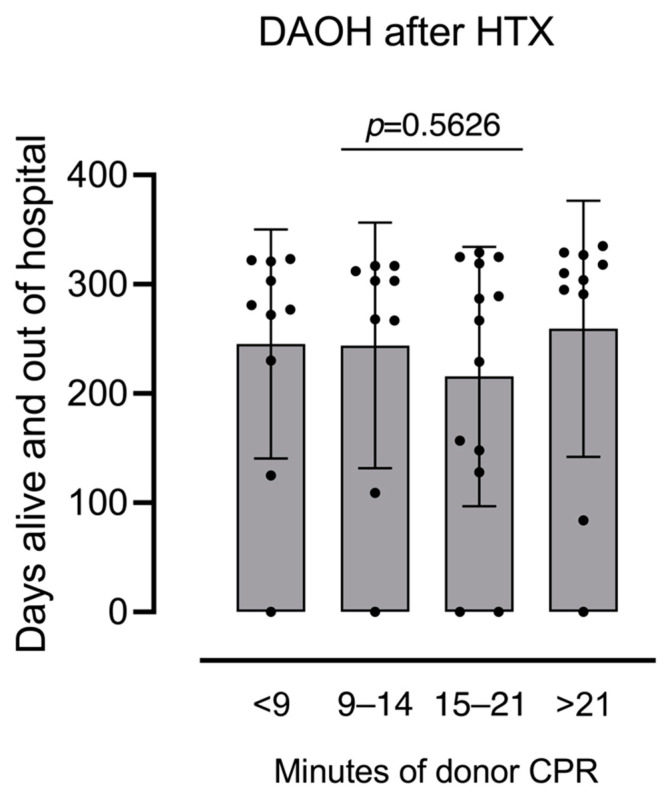
Days alive and out of hospital at one year after heart transplantation by quartile of cardiopulmonary resuscitation duration of donor hearts.

There was no significant difference in DAOH after one year between donor-CPR patients: 291 days (IQR: 211–318 days) vs. no-CPR patients: 295 days (IQR: 215–324 days; *p* = 0.619, see Figure 2). There was also no difference in DAOH when stratified by CPR duration (see Figure 3).

### 3.1. Univariate and Multivariate Linear Regression

Univariate linear regression showed that there was no association between length of CPR and DAOH (unstandardized coefficients B: −0.208, standard error: 1.42, 95% CI −3.078–2.662, *p* = 0.884). According to multivariate linear regression, the association between length of donor-CPR and DAOH was still not significant (unstandardized coefficients B: −0.06, standard error: 0.81, 95% CI −1.65–1.53, *p* = 0.943), whereas significant associations of known risk factors for low DAOH were unaffected (see Table 2).

### 3.2. Kaplan–Meier Analysis

**Figure 4:**
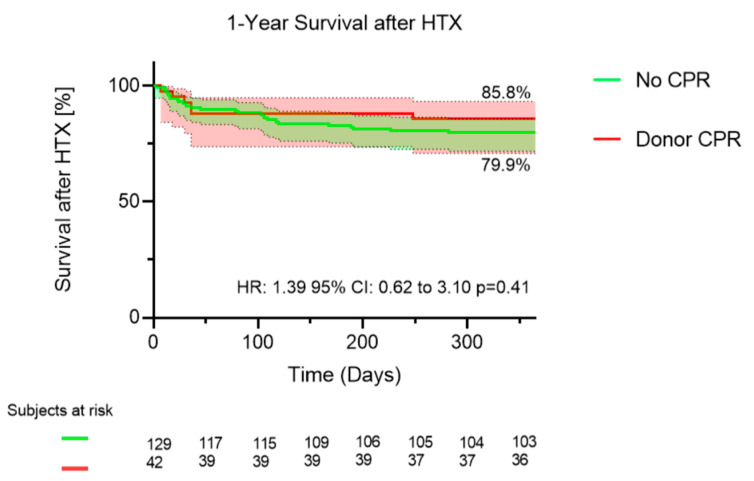
Comparison of survival at one year after heart transplantation between patients who received donor hearts with and without history of cardiopulmonary resuscitation.

Survival analysis by Kaplan–Meier method revealed that there was no significant difference between donor-CPR- and no-CPR-patients regarding survival rates at one year after HTX (donor-CPR patients = 79.9% versus no-CPR patients = 85.8%; hazard ratio = 1.39 (95% CI 0.62 to 3.10, *p* = 0.41) (see Figure 4).

## 4. Discussion

The current study aimed to analyze the impact of donor-CPR on DAOH after HTX. Our findings are in line with data on survival and suggest that a status of donor-CPR as well as the length of CPR do not negatively affect DAOH after one year.

Mehdiani and colleagues have shown in a retrospective study that postoperative morbidity and one-year mortality are not affected by CPR prior to organ donation in heart transplant patients [10]. From this, the authors drew the conclusion that donor hearts should not be rejected due to a history of CPR. Cheng et al. examined whether different durations of CPR prior to organ donation affected postoperative outcomes and survival [11]. Although a trend towards lower survival rates for longer CPR times prior to organ donation seemed to emerge from their data, this trend did not reach statistical significance.

Even earlier than that, the group around Quader and colleagues conducted a retrospective analysis of a large number of cases of HTX in the USA (*n* = 29,242, *n* = 1396 with history of CPR), reaching the conclusion that cardiac arrest and subsequent cardiopulmonary resuscitation did not induce poorer outcomes for the recipients [20]. Interestingly, a possible explanation cited by Quader et al. for why these CPR-positive hearts do not negatively affect mortality is the lack of comorbidities and generally younger age of these donors. In our cohort, donors in the donor-CPR group were not significantly younger, but were not more likely to have diabetes mellitus.

Literature on quality of life after HTX is abundant and consensually agrees that organ transplant positively affects most aspects commonly assessed in surveys (see for example, the reviews of Rosenberger et al., and more recently, Tackmann and Dettmer) [21,22]. However, such studies seldomly assess donor characteristics for their analyses and are, thus, not useful to determine the impact of CPR status of the donor on recipient QOL. To the best of our knowledge, at the time of writing this report, there are no studies comparing QOL between recipients from CPR-subjected donors vs. CPR-naive donors. We also could not find any report on DAOH for these two groups.

Seeing the scarcity of data on patient-centered outcomes, our study could further assist physicians when making choices on donor eligibility and organ allocation. Of course, clinicians primarily have to answer the question if suitable patients for HTX are able to survive when receiving a donor heart with a history of CPR. However, after successful HTX, this focus might change and factors related to functional capacity and QOL might get more and more important. From our point of view, being in hospital is not compatible with good QOL. Consequently, the number of days patients are alive and not hospitalized (=DAOH) after HTX might be an appropriate measure of long-term life impact and QOL to an extent. Referring to our data, the lack of significant difference in DAOH between our study cohorts thus might be interpreted as an additional measure of safety and suitability for CPR-positive donor hearts in regard to patient quality-of-life. Additionally, fewer days in hospital means less financial burden on healthcare systems, and DAOH might be used as a surrogate marker for healthcare costs.

### Strengths and Limitations

Our current study is subject to the usual limitations that incur for retrospective analyses. However, our center’s HTX database is collected prospectively, which can serve to ensure the quality of the data we analyzed. This study also suffers from being limited to a single center and having a modest sample size. Another limitation is the impossibility of including hospitalizations outside of our university hospital into the DAOH calculation. Although patients that underwent HTX at our center are closely connected and normally referred to us for care, we cannot exclude missing data on hospital stays, which might alter the results of our calculations.

On the other hand, the usage of DAOH as our endpoint bears the strength of including an objective quantification of QOL and healthcare costs, in addition to the standard assessment of mortality alone. A further strength of this study is the one year follow-up period.

## 5. Conclusions

With this study, we were able to show that donor CPR status and length of CPR are not associated with a reduction of DAOH at one year after HTX. Our findings emphasize the approach that CPR status might be regarded as a less important factor when deciding on donor eligibility and allocation, even for extended durations of CPR. Importantly, the results of this study should be reproduced in larger cohorts with a prospective design before final conclusions can be drawn.

## Figures and Tables

**Table 1:** Patient characteristics.

	All (*n* = 171)	Donor-CPR (*n* = 42)	No-CPR (*n* = 129)
** Baseline Characteristics of Recipients in Mean ± SD/n (%) **			
Male/female	97/74	32/10	65/64
Age (years)	54 ± 11	56 ± 10	54 ± 12
BMI (kg/m^2^)	26 ± 5	27 ± 5	25 ± 5
Creatinine (mg/dL)	1.4 ± 1.0	1.4 ± 0.7	1.4 ± 1.1
Diabetes present	34 (20)	8 (19)	26 (20)
** Baseline characteristics of donors in mean ± SD/n (%) **			
Male/female	97/74	32/10	65/64
Mismatched sex	51 (30)	6 (14)	45 (35)
Age (years)	43 ± 13	38 ± 12	44 ± 13
BMI (kg/m^2^)	26 ± 4	26 ± 5	26 ± 3
Diabetes present	11 (6)	1 (2)	10 (8)
Last dosage of norepinephrine (µg/kg/min)	0.13 ± 0.2	0.08 ± 0.08	0.14 ± 0.23
Donors with dobutamine	20 (12)	9 (21)	11 (9)
Last dosage of dobutamine (µg/kg/min)	3.51 ± 1.42	3.32 ± 0.81	3.67 ± 1.75
** Preoperative morbidities **			
Requirement of LVAD	88 (51)	23 (55)	65 (50)
Arterial hypertension	102 (60)	31 (74)	71 (55)
Pulmonal hypertension	18 (11)	5 (12)	13 (10)
Previous cardiothoracic surgeries	110 (64)	30 (71)	80 (62)
CMV IgG present	83 (49)	18 (43)	65 (50)
** Intraoperative conditions **			
total ischemic time (min)	219 ± 52	219 ± 40	219 ± 55
** Postoperative conditions **			
Dialysis	100 (58)	26 (62)	
VA-ECMO	51 (30)	15 (36)	36 (28)
Assisted ventilation (h)	151 ± 194	177 ± 207	142 ± 188
** Underlying diseases requiring HTX **			
DCM	91 (53)	19 (45)	72 (56)
ICM	67 (40)	19 (45)	48 (37)
HCM	3 (2)	1 (2)	2 (2)
ARVCM	6 (4)	1 (2)	5 (4)
Others	4 (2)	2 (5)	2 (2)
** Endpoints **			
DAOH	295 (206, 322)	291 (211, 318)	295 (215, 324)

BMI = Body mass index, LVAD = left ventricular assist device, DCM = dilated cardiomyopathy, ICM = ischemic cardiomyopathy, HCM = hypertrophic cardiomyopathy, ARVCM = arrhythmogenic right ventricular cardiomyopathy, VA-ECMO = veno-arterial extracorporeal membrane oxygenation, DAOH = days alive and out of hospital.

**Table 2:** Multivariate linear regression for the association between length of donor-CPR and DAOH at one year after heart transplantation.

Variables	Unstandardized B	Std. Error	Standardized Beta	Lower Bound 95% CI	Upper Bound 95% CI	*p*-Value
Donor age	−2.26	0.61	−0.25	−3.47	−1.05	<0.0001
Length of mechanical ventilation	−0.23	0.04	−0.38	−0.32	−0.14	<0.0001
Postoperative RRT	−50.84	17.18	0.21	−84.76	−16.91	0.004
Length of Donor CPR	−0.06	0.81	−0.005	−1.65	1.53	0.943

Std = Standard; CI = Confidence Interval; RRT = Renal Replacement Therapy; CPR = Cardiopulmonary Resuscitation.

## Data Availability

All relevant data for the understanding and interpretation of this study are included in the present manuscript.
